# Use of acceptable daily intake (ADI) as a health-based benchmark in nutrition research studies that consider the safety of low-calorie sweeteners (LCS): a systematic map

**DOI:** 10.1186/s12889-021-10934-2

**Published:** 2021-05-20

**Authors:** Seneca E. Fitch, Lauren E. Payne, Jennifer L. G. van de Ligt, Candace Doepker, Deepa Handu, Samuel M. Cohen, Njwen Anyangwe, Daniele Wikoff

**Affiliations:** 1 ToxStrategies, 23501 Cinco Ranch Blvd, Suite B226, Katy, TX 77494 USA; 2 ToxStrategies, 31 College Place, Suite B118, Asheville, NC 28801 USA; 3 College of Veterinary Medicine, University of Minnesota, 1365 Gortner Ave, St Paul, MN 55108 USA; 4 ToxStrategies, 187 Pavilion Pkwy #223, Newport, KY 41071 USA; 5 Evidence Analysis Center, Academy of Nutrition and Dietetics, 120 S. Riverside Plaza, Suite 2190, Chicago, IL 60606 USA; 6 Department of Pathology and Microbiology, University of Nebraska Medical Center, 985900 Nebraska Medical Center, Omaha, NE USA; 7 Center for Food Safety and Applied Nutrition, U.S. Food and Drug Administration, 5001 Campus Drive, College Park, MD 20740 USA

**Keywords:** Acceptable daily intake, Low-calorie sweetener, Systematic map, Scoping review, Estimated daily intake

## Abstract

**Background:**

It is well-recognized that consumers face many challenges in understanding and applying nutritional guidance for low-calorie sweeteners (LCS). Thus, this research aims to (1) assess how benchmarks for safe levels of consumption of LCS are utilized by researchers, and (2) understand how varying use of such benchmarks may contribute to challenges in understanding and applying nutritional guidance for LCS consumption.

**Methods:**

A systematic mapping exercise was employed to characterize when and how acceptable daily intake (ADI) values are used as health-based benchmarks in nutrition research studies that consider the safety of LCS.

**Results:**

Based on results from charting 121 studies, our findings demonstrate that comparisons of LCS intake to an ADI derived by an authoritative body have been made in a diverse set of published literature, varying widely in their objectives, approaches, and populations of interest. The majority of studies compared the ADI to intake in a population under study; these represent the type of comparison that is most consistent with the intent of the ADI. Other applications of the ADI included use as a benchmark in experimental studies, risk-benefit analyses, and metabolism studies.

**Conclusion:**

Although most instances of ADI use were reasonable within the context of the individual studies’ objectives, the diversity in use by original-study authors amplifies the continued need for development of “best practices” regarding the use and interpretation of the ADIs in current research. Using comparisons to the ADI can be a helpful way to provide context to research findings. However, in doing so, it is important that researchers utilize the value in a manner specific with its intent, as the ADI is a metric that represents an estimate of the amount of a substance that can be consumed daily over a lifetime without presenting an appreciable risk to health.

**Supplementary Information:**

The online version contains supplementary material available at 10.1186/s12889-021-10934-2.

## Background

Low-calorie sweeteners (LCS) are commonly defined as food additive substances used to impart a sweet taste to foods or in table-top sweeteners [1, 2]. Due to their no−/low-calorie properties and high level of sweetness relative to sugar, they are often used as sugar alternatives in foods, beverages, and medications [3]. Sugar-intake reduction and replacement is a recognized challenge in nutrition as related to associations with overweight, diabetes, and other non-communicable diseases. LCS have global importance as one of the few viable tools available to food developers to address these issues.

Fundamental to assessing the safety of LCS (or other food additives), regulatory and authoritative bodies rely on determining a safety benchmark, the “acceptable daily intake” (ADI). The ADI is defined as the estimated amount of a substance, expressed on a body-weight basis, that can be ingested daily over the course of a person’s entire lifetime without appreciable health risk; importantly, this metric is applicable to the general population, including all age ranges and physiological states [2, 4–6]. This includes children (often defined as 12 weeks and older), teenagers, adults, elderly, and pregnant and lactating women [6]*.*

ADIs are developed by scientists following extensive and critical reviews of experimental testing data. These reviews include the evaluation of genotoxicity, metabolism and pharmacokinetics, short-term toxicity, subchronic toxicity, reproductive toxicity, chronic toxicity, and carcinogenicity. Such data are often based on experimental animal studies in rodents and sometimes non-rodents, but may also include safety information from human study designs such as controlled trials or observational studies. Following review of the available battery of toxicity testing, an exposure level associated with no adverse effects (e.g., a no-observed-adverse-effect level [NOAEL]) from a study in experimental animals, expressed as a daily intake (e.g., mg ingredient/kg body weight/day) is selected. The NOAEL (or similar metric) is then divided by any applicable safety factors, resulting in the ADI. These safety factors typically represent uncertainty related to relying on experimental animal models (10x) and interindividual variability in the human population (10x). The composite safety factor is typically 100 for food additives, although it may be larger or smaller depending on the individual assessment [6].

ADI values have been established for a number of LCS (See Supplemental Table 1). The values are specific to individual sweeteners and represent an overall intake of such; as described above, these ADIs are the result of a comprehensive scientific review of an evidence base specific to the LCS, and thus, the ADI values are different. For this reason, while ST1 also shows the number of table-top sweetener packets to provide context for the ADI, the sources of consumption may vary by individual due to the wide variety of foods and beverages containing sweeteners. As with other food additives, LCS are subject to pre-market authorization assessments which determine whether the LCS is safe for consumption in the given food or beverage. Importantly, these are undertaken to assess the overall safety and determine the safe level for long-term daily consumption. While different regulatory agencies—such as the U.S. Food & Drug Administration (FDA), European Food Safety Authority (EFSA), Joint FAO/WHO Expert Committee on Food Additives (JECFA), and Scientific Committee on Food (SCF)—may derive different ADIs for a specific food additive based on the available scientific data reviewed, the general tenet required prior to market authorization is the same. That is, that a review of the scientific data by chemists, toxicologists, and other scientific experts indicates that the food additives show a “reasonable certainty of no harm” or “no apparent risk” under its conditions of intended use [4, 6, 7]. These evaluations rely on the ADI in determining whether the probable intake, often referred to as the estimated daily intake (EDI) or cumulative estimated daily intake (CEDI), is likely to be without harm. Typically, the EDI or CEDI is compared to the ADI, and if the EDI or CEDI is less than the ADI, there is reasonable certainty of no harm from consuming the food additive under its intended conditions of use. Conversely, if the EDI or CEDI is greater than the ADI, then consuming the food additive is considered not safe. This comparison is also measured as a ratio between the EDI and the ADI. If the ratio is < 1, it means that the estimated intake is lower than the acceptable intake—i.e., no risk. That is, there is some assurance that set safety levels would not be exceeded by any group of consumers, including those with the highest intake. Conversely, if the ratio is > 1, the potential for health risk is signaled, and additional evaluation is often conducted or the LCS is not approved [6–8].

Despite extensive efforts by regulators and risk assessors to gauge the safety of LCS, conflicting position statements by various associations may cause confusion among health professionals and consumers who are seeking to understand safe consumption of LCS. For example, the recently published Dietary Guidelines for Americans 2020–2025 state that while “replacing added sugars with low- and no-calorie sweeteners may reduce calorie intake in the short-term and aid in weight management, yet questions remain about their effectiveness as a long-term weight management strategy” [9]. Additionally, the guidelines state LCS are not recommended for children under the age of 2. Similar statements were made by the American Heart Association (AHA) in a recently issued an advisory that recommends against prolonged consumption of LCS beverages by children [10], with the exception of children with diabetes mellitus who may occasionally substitute LCS beverages for sugar-sweetened beverages (SSBs) when needed. AHA also advises that adults who consume high levels of SSBs may benefit from replacing these with LCS beverages to reduce intake. The National Academy of Medicine, while acknowledging the extensive FDA safety evaluation and approval process, also cautions against use by children, based on the uncertainty of effects from long-term use and low-level exposures on the health and development of children [11]. Conversely, a recent policy statement issued by the American Academy of Pediatrics says that some children, such as those with obesity and diabetes, may benefit from the substitution of sugar with LCS, although the limited amount of data on such pediatric populations is acknowledged [12]. Likewise, the Academy of Nutrition and Dietetics states that LCS are safe when consumed within the context of current dietary and physical activity recommendations for the public [13].

Thus, as a means of characterizing and addressing the potential translational needs regarding the use of ADI values by consumers and practitioners alike, the objective herein is to systematically map when and how ADI values are used in nutrition research studies that consider the safety of LCS. To achieve this, a systematic mapping approach based on the Joanna Briggs Institute (JBI) scoping review framework [14] was used to characterize the landscape of the topic in the peer-reviewed literature. By collating this type of information in the context of the direct application of ADI values, it is anticipated that the output will be informative to researchers and clinicians alike, aiding a better understanding of what types of comparisons are made in the literature, as well as what is meant by an ADI and how it can be used in safety comparisons when evaluating LCS intakes.

## Methods

The conduct of this investigation followed a stage-by-stage framework defined a priori, based on methods described by JBI [14] and James et al. [15] to develop a systematic map (also referred to as a scoping review). The research question, “In nutrition research studies, how are daily intake levels of low-calorie sweeteners in human populations assessed in the context of acceptable daily intakes (ADIs) derived by authoritative bodies?” was addressed using methods described in a protocol registered prospectively with the Open Science Framework (OSF) [16]. The methods are summarized here. The protocol also provides descriptions of the roles of all of the research team members, including the advisory panel members, each of whom has specific subject matter expertise engaged specifically to provide guidance and ensure quality, integrity, and comprehensiveness in the research. In doing so, these researchers were also involved with the conduct of the work, including providing input and approval for the search strategy, selection of studies, and analyses, as well as reviewing and revising this manuscript.

The Population, Concept, and Context (PCC) elements were used to define inclusion criteria:

### Population

Studies that included humans of any health status, age, and sex; including sensitive and healthy populations, pregnant or nursing women, and all geographies, regions, races, and ethnicities.

### Concept

Studies making any type of comparison of an established ADI value to a measured or estimated daily intake of relevant LCS were included as follows:

- LCS included acesulfame-K, advantame, alitame, aspartame, cyclamate, d-tagatose, monk fruit, neotame, steviols (and stevia extracts), saccharin, and sucralose (alone or in combination).
- Intakes reported as a quantitative value, based on a controlled exposure; cumulative or specific food intakes; or blood, serum, or urinary concentrations.
- Studies must have made an original comparison of a designated ADI (by any authoritative body) to the included.

### Context

This review considered any study that evaluated the intake, health benefits and/or risk, or safety of LCS consumption. The concept under evaluation—safe levels of LCS consumption, as defined by ADI values established by authoritative bodies—implicitly contains outcomes related to safety.

### Types of studies

Peer-reviewed publications describing epidemiological, clinical, or laboratory settings, including primary research and secondary (e.g., reviews) and case studies/reports were considered; non-peer-reviewed authoritative reports were also included. Publications that were not available in English were noted but not included in mapping.

#### Search strategy

The literature search strategy was developed by an Information Specialist, informed by input from stakeholders (ILSI LCS Committee), and reviewed by the Advisory Panel. Specifically, stakeholders provided LCS identifiers (e.g., trade names, synonyms) to be used in the development of the search syntax. As described in the protocol, database-specific search syntax was developed for PubMed and Embase citation databases. Syntax included terms targeting LCS (e.g., CAS numbers and trade names), and terms related to intake (e.g., food frequency, consumption), exposure, and safety (e.g., acceptable daily intake). The protocol, including the full electronic search strategy, is available as Supplemental Material.

Publications were also identified via a series of discussions with stakeholders and Advisory Panel Members. Specifically, the ILSI North America LCS Committee and Advisory Panel Members provided input in the form of background literature as experts in LCS research. This literature was reviewed for context and used in validation exercises of database searches. Supplementary to the traditional citation database searching, hand-searching and reference harvesting were also implemented by reviewing titles in the citation lists of relevant publications. When relevant titles were identified, these citations were added to the literature screening process for comparison to eligibility criteria. Targeted searching of FDA, EFSA, and JECFA websites and ToxPlanet was also performed to obtain authoritative documents related to the derivation of individual LCS ADIs.

#### Study selection

Search results were de-duplicated via EndNote X9 (Clarivate Analytics, PA, USA) and uploaded to the systematic review software DistillerSR (Evidence Partners, Ontario, CA) for both title and abstract (TiAb) screening and full-text review. Piloting of TiAb screening and full-text review were performed; TiAb screening was performed by two reviewers. Studies meeting all inclusion criteria were advanced to full-text review. Full-text review was performed by a single reviewer, and publications excluded at this stage were subject to a 100% quality control (QC) screening by a second reviewer; any resulting inclusion/exclusion conflicts were resolved by discussion between reviewers. Screening and study selection was completed by in full by S.F., L.P., and D.W.

#### Data extraction and mapping

For data extraction (or charting), templates were developed in DistillerSR based on the JBI framework [14] and included information key to informing the research objective. Following development of these templates, a second reviewer calibration effort was implemented to ensure reviewer consistency. These pilot exercises resulted in iterative refinement of the DistillerSR form to increase clarity and collect additional data determined to be useful to informing the analysis.

During the pilot of the charting template and workflow, it became evident to the research team that further criteria needed to be developed to account for such a heterogeneous body of literature. As a solution, publications were evaluated for their relevance to the objective and research question, and subsequently were categorized as “directly relevant” or “contextual.” This separation also prevented collection of duplicate data, as would be the case in a scenario where published empirical data were included in a review, and both publications were identified for inclusion in this investigation. In this assessment, “directly relevant” is to be interpreted as a “*comparison of an original intake estimate or controlled exposure to an ADI as derived by an authoritative body (i.e., primary research),*” while “contextual” is to be interpreted as “*informing part, but not all, of the research question and comprising secondary research, such as reviews of intake studies, safety data, or ADI derivation.”* During charting, this designation determined the type of information collected for each, an overview of which is provided in Table 1.

**Table 1:** Data fields collected during charting of included studies classified as directly relevant or contextual

Collected data	Direct	Contextual
Reference ID	✓	✓
Author	✓	✓
Title	✓	✓
Year	✓	✓
Study category	✓	
Objective	✓	✓
Population description	✓	
Is this a sensitive population?	✓	
Brief method for measuring intake	✓	
LCS of interest	✓	✓
ADI value used in comparison	✓	✓
ADI to LCS intake comparison	✓	✓
Geographic region of study	✓	
Authoritative body referenced	✓	
Conclusion of research summary	✓	
Author recommendations	✓	
Author-reported strengths or limitations	✓	
Funding sources	✓	
References for hand-searching	✓	✓
Additional notes or comments	✓	✓

Charting was performed by a single reviewer (S.F. or L.P), and authors were not contacted if information was not reported in the publication. All information from DistillerSR was exported to Microsoft Excel to develop tabular summaries and map the charted data, in order to characterize the body of literature.

## Results

### Study identification and selection

**Fig. 1:**
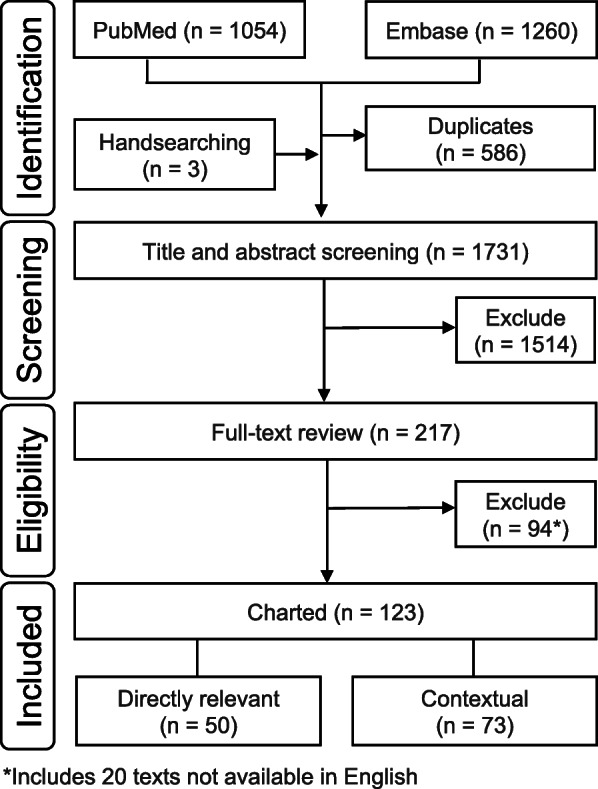
Flow chart of literature identification, screening, and categorization results

During syntax development, search validation was performed to inform the preciseness and sensitivity of the search strategy. Validation studies identified by stakeholders (*n* = 19) were compared to the results, and syntax was revised to include key terms of publications that were not retrieved. Revisions to syntax during this effort increased retrieval from 58% (11/19) to 89% (17/19), and based on this, a broad-concept search (limited to human populations), supplemented by a narrow, targeted search, was selected. The literature searches were performed in the PubMed and Embase databases on January 16, 2019, with no date restrictions. TiAb review and full-text screening were performed from January 2019 to April 2019. In total, 121 publications were included in the systematic map; Fig. 1 illustrates the study numbers of the process at each stage. Studies excluded during TiAb fell into one or more of four categories: (1) no intake estimated or measured; (2) irrelevant population, such as experimental animals; (3) LCS not assessed; (4) no relation to the PCC (e.g., analytical wastewater quantitation). At the full-text review stage, studies were also excluded for (1) lacking comparison of an ADI to an estimated intake; (2) lack of full-text availability (including conference abstracts); and (3) not available in English.

### Study characteristics and publication patterns

**Fig. 2:**
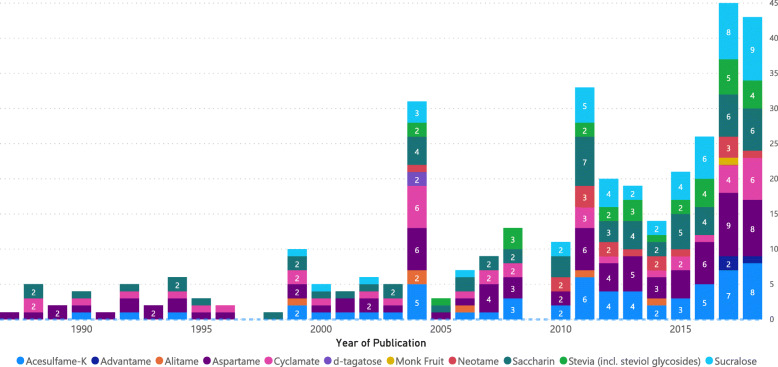
Number of included publications per LCS sweetener by year of publication

A publication frequency chart visualizing the number of publications per year, by individual LCS, is shown in Fig. 2. In general, the number of publications per year increases from 1987 to the present; more than half (55%) of the identified studies were published in the final 8 years (2011 to 2018) of the range, compared to 45% published in the first 24 years of the range (1987 to 2010).

The overall occurrences of the LCS of interest in publications are described in Table 2. Of the LCS evaluated herein, aspartame intake was the first to be compared to the ADI (1987) [17], and was compared most frequently (*n* = 82); advantame and d-tagatose accounted for the least often compared. When considering only directly relevant studies, advantame, alitame, d-tagatose, and monk fruit intakes have not been compared to an ADI. This is reasonable based on the lack of established ADI values for the latter three.

**Table 2:** Number of publications evaluating each LCS of interest by level of relevance

	Direct	Contextual	Total
	No. of publications		
**LCS of interest (first pub. year)**			
Acesulfame (1990)	30	29	59 (17%)
Advantame (2017)	0	2	2 (1%)
Alitame (1999)	0	6	6 (2%)
Aspartame (1987)	33	49	82 (24%)
Cyclamate (1988)	22	19	41 (12%)
d-Tagatose (2004)	0	2	2 (1%)
Monk fruit (2013)	0	3	3 (1%)
Neotame (2004)	2	13	15 (4%)
Steviols (2004)	10	19	29 (8%)
Saccharin (1988)	29	36	65 (18%)
Sucralose (1999)	16	32	48 (14%)

### Use of ADI within the evidence base

**Fig. 3:**
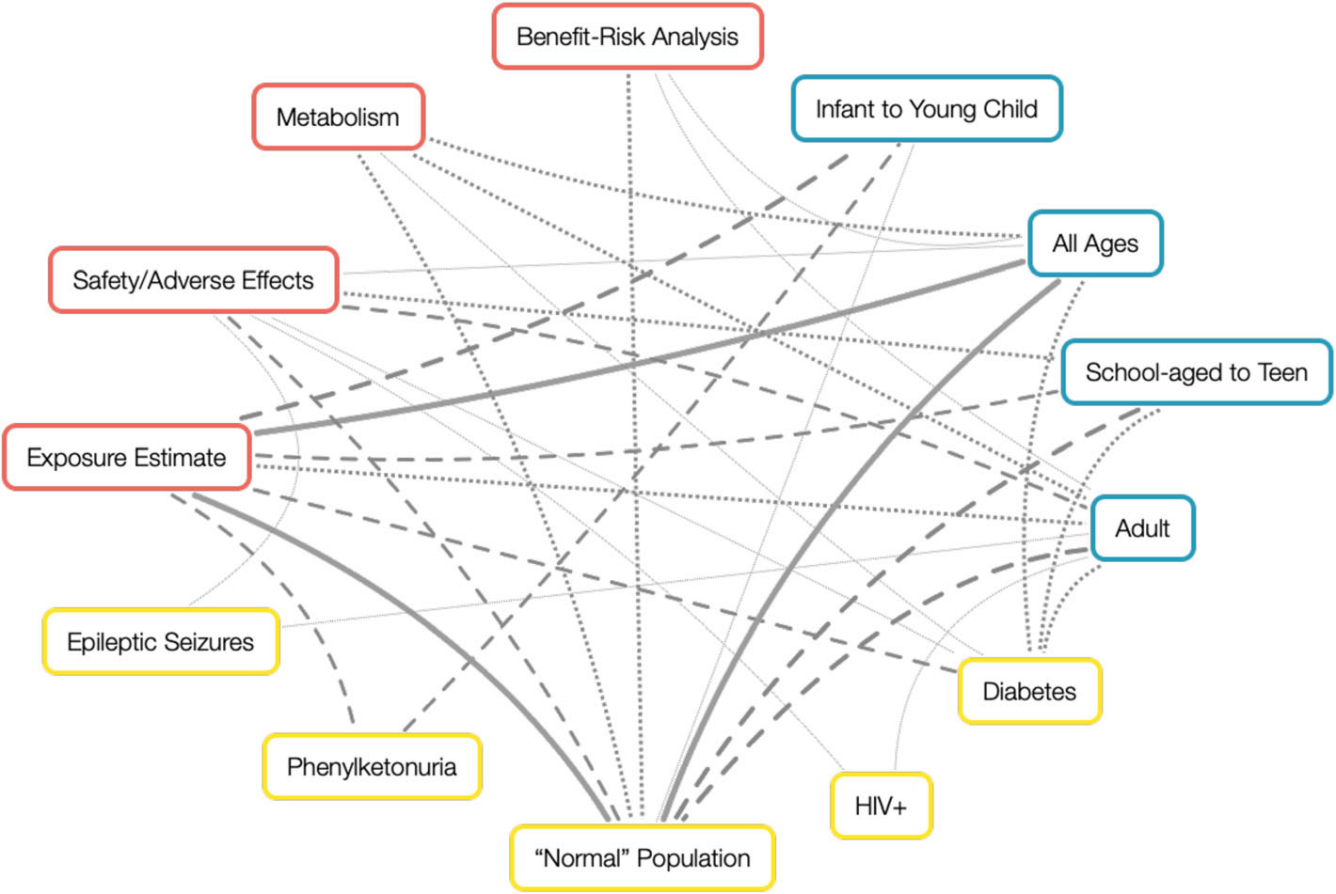
Relationship map of population health status (yellow), age group (blue), and research study type (red). Line weights indicate the number of studies that evaluated each sub-topic

**Fig. 4:**
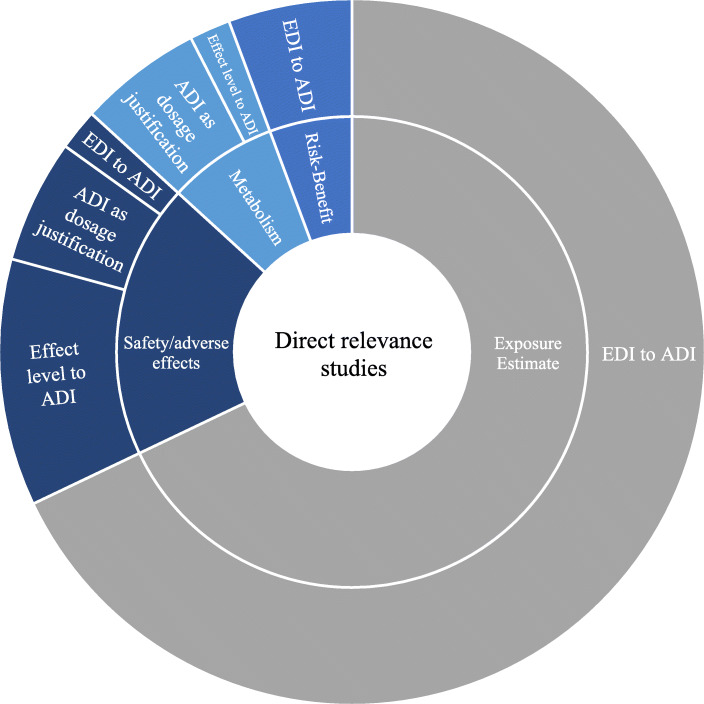
Distribution of study categories by research goals and their respective use of an ADI for comparison to an LCS intake

Direct studies were mapped to four categories based on charted information, to characterize the body of literature in which LCS intakes are compared to ADIs: (1) the overall goal of the study (i.e., research type), (2) the health status of the population of interest, (3) the population’s age, and (4) the context of the LCS intake-to-ADI comparison (Figs. 3 and 4). Relationships between several of these categories are depicted in Fig. 3. If considering the most-studied relationships (i.e., a knowledge cluster) in the context of this evaluation, it is apparent that exposure estimates within a normal population of all ages have been studied most extensively. In contrast, studies evaluating intake relative to the ADI in infant populations, for example, are less frequently available. The subsequent sections further characterize the evidence base as related to ADI usage.

#### Research types (goals of studies)

Major themes for the overall goal of the publicly available research include intake/exposure estimation (EE), evaluation of safety or adverse effects (AE), risk-benefit analysis (RBA), or the assessment of LCS metabolism (Met). EE studies account for the majority of the database (68%) and sought to estimate LCS exposure by assessing consumption and sweetener concentrations to model probable intake levels for a population of interest. Consumption was assessed by a variety of methods, including food frequency questionnaires, national consumption databases, and import/export statistics. LCS concentrations were generally based on maximum permissible limits of food and beverage categories, analytical sampling, and substitution via sucrose equivalents. These components were then used to calculate a probable intake, or EDI, of a specific LCS. Publications reporting intakes derived using these methods, and comparing them to an ADI, are included for acesulfame, aspartame, cyclamate, neotame, steviols, saccharin, and sucralose to an ADI. No EE studies that included intakes for advantame, alitame, d-tagatose, or monk fruit were identified. In AE studies (19%), calculated intakes were based on controlled LCS administration (i.e., clinical trials), or were estimated for normal food and beverage consumption using methods similar to that of an EE. This type of study was identified in the comparisons of acesulfame, aspartame, cyclamate, steviols, and sucralose intake to an ADI. LCS intake values reported in Met studies (8%) were composed of controlled exposures, to identify urinary biomarkers following intake, to investigate rates of metabolism, or to determine differences between adults and children in LCS blood concentrations. Intakes compared to an ADI in this category were identified for acesulfame, cyclamate, steviols, saccharin, and sucralose. In the three RBAs (6%), investigators examined the intake of added sugars or sucrose in relation to health hazards compared to the potential health risks of LCS substitution. LCS intake values based on both controlled and estimated exposure methods were reported for acesulfame, aspartame, cyclamate, and saccharin.

#### Health status and age

Health status of the studied populations was categorized largely as “normal” populations (74%), which includes studies that recruited participants from the general public or those based on population-level statistics. Inclusion of pregnant and breastfeeding women in these studies varied, but no studies were identified that evaluated this sub-population specifically. Other health statuses, characterized herein as sensitive populations, include diabetes (11%), phenylketonuria (8%), epilepsy (4%), HIV+ (2%), and overweight (2%). These sensitive groups were considered populations of interest based on the likelihood of increased LCS intake or potential differences in the effects of LCS, compared to “normal” humans. The age range of the database spans all life stages, and study populations were categorized into four groups: infants to young children (9%), school-aged children to teenagers (19%), adults only (25%), and all ages included (47%). In the case that a study population spanned two of these categories, it was placed in whichever range included the majority of the participant ages.

#### LCS:ADI comparison

Comparisons of LCS intake to an ADI within directly relevant studies were categorized to better understand their use. These categories were based on the types of comparison and include: ADI as a safety benchmark to determine an appropriate dosage in controlled exposure studies, margin-of-safety (MOS) calculations (EDI compared to ADI), and effect (or no-effect) levels compared to an ADI to discuss safety (Supplementary Tables 1, 2, and 3, respectively). The majority of the database studies are those using an EDI/ADI ratio to investigate safety in a population of interest (75%). This type of assessment is standard in determining the likelihood of an exceedance and is similar to the method used by regulatory agencies during food-additive evaluations [18]. While limitations to these studies exist and are reported by authors (relating to the specific methods or surveys employed in the individual studies), it is standard to utilize the data collected from such surveys (e.g., consumption for one or 2 days) across a population to represent intake for the respective age group or geography, etc., as evaluated in the given study (i.e., the estimated daily intake, or EDI). The EDI estimates resulting from these studies are generally accepted to represent intake over period of time. For this reason, the use of this type of ADI comparison is appropriate. Though beyond the scope of this assessment, it is recognized that an average estimate of intake over a lifetime may differ from the intake estimated for specific age groups, such as children and teenagers. A MOS may be less in these sub-populations relative to adults, even if consumption is similar, simply because both the EDI and ADI metrics are bodyweight adjusted, and the body weights of children and teenagers are lower than those of adults. The potential for short-term exceedances during some life stages (e.g., exceedance of an ADI for children on special diets) has been discussed by Martyn et al. [19]; exceedance of an ADI for a fraction of a lifetime may not indicate a safety concern, given the low acute toxicity potential combined with the safety factors inherent to development of the ADI itself.

The vast majority of the studies that compared ADIs to EDIs concluded with an acceptable MOS (i.e., no concern). For example, Martyn et al. [20] estimated dietary intakes of four artificial sweeteners (acesulfame K, aspartame, saccharin, and sucralose) by Irish pre-school children aged 1–4 using multiple sources (food consumption data from a 4-day national survey, LCS presence probability, LCS concentration data, and maximum permitted levels of LCS) and compared to the ADI for each LCS; the authors concluded a lack of safety concerns based on this comparison. DeWinter et al. [21] used a food frequency questionnaire to assess intake of acesulfame-k, aspartame, neohesperidin, sucralose, saccharin, steviol glycosides, and neotame in type 1 diabetic children, concluding that there is little chance to exceed the ADI of each LCS. Alternatively, in a large population-based dietary survey conducted by Vin et al. [22], the authors reported on dietary exposure of 13 priority additives in four European countries (France, Italy, the UK, and Ireland), finding a lack of concern for the LCS evaluated based on comparisons to the ADI (intake less than the ADI) for all ages evaluated (1 to > 97). Although these studies evaluated different populations, ages, and LCS, and also used different methods to estimate intake of LCS, the comparisons of EDI to ADI were conducted in a manner consistent with the intended purpose of the ADI.

Comparisons of dosage levels (11%) and effect (or no-effect) levels (13%) to an ADI account for the remainder of the database. In the first type, studies used the ADI as a safety benchmark to either justify or determine an appropriate intake for the LCS of interest. For example, in evaluating cyclamate metabolism in a diabetic population, Buss et al. [23] state that “a daily dose of 1g of cyclamate was chosen since this slightly exceeds the ADI for the sweetener (0–11 mg/kg/day) established by the JECFA and SCF.” The last of these categories (i.e., effect-level comparisons), is composed of studies that related the intake at which an effect was observed to the ADI. For instance, Kim et al. [24] makes this type of comparison in an evaluation of potential associations between eating habits (including intakes of LCS) and the emotional states of school children. The authors stated that mean LCS intake of school-age girls was < 1% of the ADI and correlated positively with electroencephalograph theta-beta ratios that were used as a proxy for attention, emotional regulation, or resilience against stress.

## Discussion

This mapping exercise identified, charted, and characterized instances of LCS intakes compared to ADIs in the published literature. The EE studies represent both the largest portion of the evidence base herein and those most consistent with the directed use of ADIs (i.e., that which is standard practice in determining a MOS). These included studies that compared intakes in specific populations (e.g., specific age group or health status, geography, etc.) and general populations for each of the LCS mapped herein. Though composing a smaller portion of the evidence base, RBA studies were similar, because the risk of an outcome associated with sugar intake was compared to the heightened level of LCS consumption due to substitution; this increased LCS intake was then compared to the ADI to assess any potential risks. The remainder of the evidence base, Met and AE studies, used the ADI in a manner that was reasonable within the context of the individual study’s research objectives (e.g., comparison of a no-effect level or dosage justification), although such applications do not represent standard uses of the ADI.

Overall, the study findings across the evidence base were diverse in nature, as expected from such a heterogeneous data set. This range of findings highlights the complexity of these studies evaluating multiple populations, LCS, intake scenarios, and other elements. For example, among the EE studies, both risk and no risk of exceeding the ADI were reported—in many cases, varying within the same study depending on the scenarios used for the model exposures [25–27]. Reporting the risk of exceeding an ADI (as in these cases), however, is not synonymous to reporting the risk of adverse health effects. Rather, these considerations are more often used inform regulations and food standards specific to certain populations. Further assessment of such data to reach a conclusion about these risks would be more appropriate for evaluation under systematic review. However, it is of interest that, in charting the use of the ADI, there was a large amount of variability in how the results of LCS EDI-to-ADI comparisons were translated into conclusions or recommendations made by authors when addressing safety or health risks. Some authors concluded or recommended that additional research was needed, maximum permissible limits should be reduced, ADI values should be reassessed, or modifications should be made to dietary habits, even when EDIs were below ADIs. Such conclusions and recommendations were typically expressed in the discussion section of the studies reviewed and were those proffered by the authors. Similarly, in AE studies, conclusions or recommendations regarding (or questioning) the protective nature of the ADI values were offered by authors based on their study findings without considering the context of the development of the ADI—and specifically, without rigorously investigating the given endpoint as it was evaluated as part of the ADI development.

As a result, the studies that use non-traditional applications of the ADI, as well as EE studies that do not consider a representative LCS daily intake evaluate (vs. single-day exceedances, for example) should be interpreted with caution by the reader. This may include consideration of the individual study relative to the overall body of evidence—which may involve context of a particular effect relative to the database evaluated as part of ADI development, the conditions of the pivotal study from which the ADI was developed, the pharmacokinetic parameters, or perhaps context of exposure in a particular population or person relative to longer term exposure. Such a concept has been recognized for decades [28], yet is not practiced consistently in the studies charted herein.

It is equally important to understand the complexities in evaluating exposure in order to compare to the ADI Martyn et al. [19]. There is uncertainty in estimating exposure in a person, or in a population, which depends on both the concentration or amount of an LCS in a food or beverage (e.g., how much of a specific LCS in a certain food type) and the amount of the food or beverage consumed. Compounding this uncertainty is the lack of food consumption information in many developing countries, which can present significant challenges for accurate dietary intake studies therein. Thus, evaluation of exposure involves estimation and/or modeling in some capacity, which can range from simple deterministic estimations to more complex, probabilistic methods. Due to the potential for extensive variability, a recent review by Martyn et al. [19] called for a standardization of approaches to better improve the science, particularly related to changes in exposure events such as sugar reduction recommendations. This was recognized as especially important for high-risk individuals, including people with diabetes and children with specific dietary requirements. These elements represent the critical nature of characterizing exposure in a way that is amenable for comparison to the ADI.

With respect to the mapping of ADI use in this research, limitations are inherent to the broad scope and nature of the research question. For example, while our search strategy was performed systematically (a strength), it is possible that more papers exist than were identified due to a likelihood that LCS intake is compared to an ADI in the discussion or conclusions, which is not always captured by traditional searches. To address this problem, additional hand-searching was performed using reference lists of relevant publications and searching full texts for key terms where possible. Additionally, studies that were excluded due to being published in a foreign language could add additional insight to the context of comparisons and geographic regions where research is taking place. Based on a review of English titles and abstracts of references in this exclusion category, study populations in Brazil, Chile, Latin America (general), Mexico, and Russia have also been evaluated. Further strengths include that an a priori protocol was registered on a publicly accessible data repository, and that an Advisory Panel with diverse expertise was also engaged to provide guidance and ensure quality, integrity, and comprehensiveness in the research.

### Implications for practitioners

From the practitioner’s perspective, it is important to maintain the understanding that an ADI is defined as the amount of a substance, expressed on a body-weight basis, that can be consumed daily over the course of a person’s entire lifetime without appreciable health risk, and is applicable to the general population, including all age ranges and physiological states (including children and pregnant women). Thus, when interpreting findings from population-based consumption surveys, or even when characterizing an individual’s consumption of LCS, the ADI is a safe level of consumption derived by regulatory scientists from a database of studies specific to the individual LCS. Moreover, the ADI accounts for inherent uncertainty via safety factors, and thus is not viewed as a “bright line”—it is not intended to serve as a threshold for safety at one moment or period in time [6–8]. Thus, single exceedances should not be viewed as having the potential for adverse effects, and the context around the use of an ADI is critical relative to its use.

## Conclusion

Using a systematic mapping approach, our findings demonstrate that comparisons of LCS intake to an ADI derived by an authoritative body have been made in a diverse set of published literature, varying widely in their objectives, methods used, and populations of interest. While most instances of ADI comparisons are based on a reasonable approach in the context of individual studies’ objectives, it is important that LCS intake be assessed and interpreted appropriately relative to ADIs developed by authoritative bodies. This is particularly important for consumers and practitioners looking to inform their decision on the use of LCS in efforts related to public health goals of lowering added sugars as part of improving health outcomes [29]. This work highlights the need for researchers to continue to adhere to responsible use of the ADI, as well as the need for continued education related to the development and use of ADI values.

## Supplementary Information

**Additional file 1: Supplemental Table 1.** LCS and their respective ADIs, as designated by the FDA, EFSA/SCF, and WHO/JECFA. **Supplemental Table 2**. Studies of direct relevance in which authors use the ADI as a safety benchmark to determine an appropriate intake for a controlled exposure. **Supplemental Table 3.** Studies of direct relevance in which authors compare an EDI to an ADI to estimate the MOS in the population of interest. **Supplemental Table 4.** Studies of direct relevance in which authors compare an effect (or no-effect) level to an ADI to discuss safety. **Supplemental Table 5**. Studies of contextual relevance. **Supplemental Table 6**. Studies excluded from mapping during full-text review.

**Additional file 2.**

## Data Availability

Data sharing is not applicable to this article as no datasets were generated or analyzed during the current study.

## Abbreviations

ADI: Acceptable daily intake
AE: Adverse effects
AHA: American Heart Association
CEDI: Cumulative estimated daily intake
EDI: Estimated daily intake
EE: Exposure estimation
EFSA: European Food Safety Authority
FDA: U.S. Food & Drug Administration
JBI: Joanna Biggs Institute
JECFA: Joint FAO/WHO Expert Committee on Food Additives
LCS: Low-calorie sweeteners
Met: Metabolism
MOS: Margin-of-safety
NOAEL: No-observed-adverse-effect level
RBA: Risk-benefit analysis
SCF: Scientific Committee on Food
SSBs: Sugar-sweetened beverages
OSF: Open Science Framework
PCC: Population, Concept, and Context
TiAb: Title and abstract

## References

[1] Union E (2019). Sugars and Sweeteners.

[2] European Food Safety Authority (2019). Sweeteners.

[3] Food and Drug Administration (2018). Additional information about high-intensity sweeteners permitted for use in food in the United States.

[4] Food and Drug Administration (2014). High-Intensity Sweeteners.

[5] IPoC S, International Programme on Chemical Safety (2004). IPCS Risk Assessment Terminology.

[6] World Health Organization (1987). Principles for the safety assessment of food additives and contaminants in food. Environmental Health Criteria.

[7] Authority EFS (2012). Guidance for submission for food additive evaluations. EFSA J.

[8] Rulis AM, Levitt JA (2009). FDA'S food ingredient approval process. Safety assurance based on scientific assessment. Regul Toxicol Pharmacol.

[9] U.S. Department of Agriculture and U.S (2020). Department of Health and Human Services. Dietary Guidelines for Americans, 2020–2025.

[10] Johnson RK, Lichtenstein AH, Anderson CAM, Carson JA, Després JP, Hu FB, Kris-Etherton PM, Otten JJ, Towfighi A, Wylie-Rosett J, American Heart Association Nutrition Committee of the Council on Lifestyle and Cardiometabolic Health; Council on Cardiovascular and Stroke Nursing; Council on Clinical Cardiology; Council on Quality of Care and Outcomes Research; and Stroke Council (2018). Low-calorie sweetened beverages and Cardiometabolic health: a science advisory from the American Heart Association. Circulation..

[11] Institute of Medicine (2007). Nutrition standards for foods in schools: leading the way toward healthier youth.

[12] Baker-Smith C, de Ferranti SD, Cochran WJ (2019). The use of nonnutritive sweeteners in children. Pediatrics..

[13] American Dietetic Association (2012). Position of the american dietetic association: use of nutritive and nonnutritive sweeteners. J Am Diet Assoc.

[14] Peters MD, Godfrey C, McInerney P, Baldini Soares C, Khalil H, Parker D (2017). Joanna briggs institute reviewer's manual: the joanna briggs Institute.

[15] James KL, Randall NP, Haddaway NR (2016). A methodology for systematic mapping in environmental sciences. Environ Evid.

[16] Fitch S, Jvd L, Payne L, Doepker C, Kleinman R, Handu D (2019). A Systematic Map of the Use of Acceptable Daily Intake (ADI) as a health-based benchmark in nutrition research studiesthat consider the safety of Low-Calorie Sweeteners (LCS).

[17] Maher TJ, Wurtman RJ (1987). Possible neurologic effects of aspartame, a widely used food additive. Environ Health Perspect.

[18] Alger HM, Maffini MV, Kulkarni NR, Bongard ED, Neltner T (2013). Perspectives on how FDA assesses exposure to food additives when evaluating their safety: workshop proceedings. Compr Rev Food Sci Food Saf.

[19] Martyn D, Darch M, Roberts A, Lee HY, Tian TY, Kaburagi N (2018). Low−/no-calorie sweeteners: a review of global intakes. Nutrients.

[20] Martyn DM, Nugent AP, McNulty BA, O’Reilly E, Tlustos C, Walton J (2016). Dietary intake of four artificial sweeteners by Irish pre-school children. Food Addit Contam.

[21] Dewinter L, Casteels K, Corthouts K, Van de Kerckhove K, Van der Vaerent K, Vanmeerbeeck K (2016). Dietary intake of non-nutritive sweeteners in type 1 diabetes mellitus children. Food Addit Contam Part A Chem Anal Control Expo Risk Assess.

[22] Vin K, Connolly A, McCaffrey T, McKevitt A, O'Mahony C, Prieto M (2013). Estimation of the dietary intake of 13 priority additives in France, Italy, the UK and Ireland as part of the FACET project. Food Addit Contam.

[23] Buss NE, Renwick AG, Donaldson KM, George CF (1992). The metabolism of cyclamate to cyclohexylamine and its cardiovascular consequences in human volunteers. Toxicol Appl Pharmacol.

[24] Kim JY, Kang HL, Kim DK, Kang SW, Park YK (2017). Eating habits and food additive intakes are associated with emotional states based on EEG and HRV in healthy Korean children and adolescents. J Am Coll Nutr.

[25] O'Sullivan AJ, O'Mahony C, Meunier L, Loveridge N, McKevitt AI (2018). Investigation of the potential for a simplified exposure tool in medical nutrition (SETIM) to minimise exposures to sweeteners in young patients aged 1-3 years with PKU and CMPA. Food Addit Contam Part A Chem Anal Control Expo Risk Assess..

[26] O’Sullivan AJ, Pigat S, O’Mahony C, Gibney MJ, McKevitt AI (2016). Probabilistic modelling to assess exposure to three artificial sweeteners of young Irish patients aged 1–3 years with PKU and CMPA. Food Addit Contam.

[27] O’Sullivan AJ, Pigat S, O’Mahony C, Gibney MJ, McKevitt AI (2017). Longitudinal modelling of the exposure of young UK patients with PKU to acesulfame K and sucralose. Food Addit Contam.

[28] Renwick AG, Walker R (1993). An analysis of the risk of exceeding the acceptable or tolerable daily intake. Regul Toxicol Pharmacol.

[29] Evert AB, Boucher JL, Cypress M, Dunbar SA, Franz MJ, Mayer-Davis EJ, Neumiller JJ, Nwankwo R, Verdi CL, Urbanski P, Yancy WS (2014). Nutrition therapy recommendations for the management of adults with diabetes. Diabetes Care.

