# Supplementary material for: Use of acceptable daily intake (ADI) as a health-based benchmark in nutrition research studies that consider the safety of low-calorie sweeteners (LCS): a systematic map
Source: BMC Public Health. 2021 May 20;21:956. doi: 10.1186/s12889-021-10934-2 (PMC8138992; doi:10.1186/s12889-021-10934-2)
Supplement: Supplementary file 2 — Additional file 2. [file 12889_2021_10934_MOESM2_ESM.pdf]

Systematic Map Protocol

Title

A Systematic Map of the Use of Acceptable Daily Intake (ADI) as a Health-Based Benchmark in Nutrition Research Studies that Consider the Safety of Low-Calorie Sweeteners (LCS)

Author Information

Seneca Fitch[1], Jennifer van de Ligt[1,2], Lauren Payne[1], Candace Doepker[1], Ronald Kleinman[3‡], Deepa Handu[4‡], Samuel M. Cohen[5‡], Njwen Anyangwe[6‡], Daniele Wikoff[1*]

[1]ToxStrategies, Inc.
[2]University of Minnesota
[3]Mass General and Harvard Medical School
[4]Academy of Nutrition and Dietetics
[5]University of Nebraska Medical Center
[6]U.S. Food and Drug Administration Center for Food Safety and Applied Nutrition
*Corresponding Author; dwikoff@toxstrategies.com
‡ Advisory Panel

Guidelines for Protocol Preparation

The protocol, as well as all subsequent efforts, will be conducted and reported in accordance with the Center for Open Science's Transparency and Openness Promotion (TOP) Guidelines[1]. Consistent with such, the protocol was developed in accordance with the Joanna Briggs Institute (JBI) Reviewers' Manual 2015 – Methodology for JBI Scoping Reviews[2]. Contained in this protocol are the elements which the JBI manual specifies that the protocol include: author information, development of the title, objective, and question, background, inclusion criteria, search strategy, extraction of the results, and presentation of the results. Additional elements, such as author roles, the funding source, disclosure of interests, are also provided herein.

Author Roles

The project will be carried out by a multidisciplinary team consisting of subject matter experts, clinicians, experts in evidence-based methodologies including systematic mapping, evidence analysts, and an information specialist. The project team includes an Advisory Panel whom have the responsibility of ensuring the quality, integrity, and

---

[1] https://cos.io/our-services/top-guidelines/
[2] http://joannabriggs.org/assets/docs/sumari/Reviewers-Manual_Methodology-for-JBI-Scoping-Reviews_2015_v1.pdf

comprehensiveness of the research. The table below describes the roles of project team members as it relates to topic formulation and protocol development as well as anticipated roles for the conduct of the systematic map and subsequent reporting.

Table 1. Author roles.

Author	***Protocol***	***Systematic Map***	***Reporting (manuscript)***
Seneca Fitch	A, D, F, I, P	A, D, F, I, P	A, D, F, I, P
Jennifer van de Ligt	A, D, I, P	A, D, I, P	A, D, I, P
Lauren Payne	A, D, I, P	A, D, I, P	A, D, I, P
Candace Doepker	A, D, I	A, D, I	A, D, I
Daniele Wikoff	A, D, F, I, P	A, D, F, I, P	A, D, F, I, P
Advisory Panel Members	A, I	A, I	A, I

A = approve, D = develop, F= facilitate, I = input, P = perform

Funding sources/sponsors

This work was commissioned by International Life Sciences Institute (ILSI) North America. ILSI North America is a public, non-profit scientific foundation that advances the understanding and application of science related to the nutritional quality and safety of the food supply. ILSI North America receives support primarily from its industry membership. The views expressed in this protocol are those of the coauthors.

Disclosure of interests

DW, JV, SF, LP and CD are employed by ToxStrategies, Inc., which is a consulting firm providing services to private and public organizations on toxicology and risk assessment issues. ToxStrategies received consulting fees for tasks related to topic formulation and protocol development. None of the authors from ToxStrategies received personal fees, nor will the ToxStrategies authors directly benefit, financially or non-financially, from the conclusions of the research task.

SC serves on the Flavor and Extract Manufacturers Association (FEMA) Expert Panel that evaluates flavor ingredients. Additionally, SC was part of a review sponsored by McNeil Nutritionals on sucralose, published in Nutrition and Cancer, 68: 1-15, 2016.

During the last 12 months, RK has been involved in the following activities described below. The board memberships are all non-profit, unpaid. The International Life Sciences Research Foundation pays for travel expenses for the 2 in-person board meetings each year.

1. MGH Physicians Organization: member Board of Trustees
2. International Life Sciences Research Foundation: member Board of Trustees
3. David Ortiz Childrens Fund: member Board of Trustees
4. UNICEF New England: member Board of Trustees
5. Global Child Nutrition Foundation: Chair and member Board of Trustees
6. Project Bread, Boston: Chair and member Board of Trustees
7. European Biomedical Research Institute, Salerno (EBRIS): member Board of Trustees
8. Sesame Street Foundation: Advisor, Children's Nutrition
9. General Mills Scientific Advisory Board 2 days./year (honorarium)
10. American Academy of Pediatrics, Editor, Pediatric Nutrition, 8th Edition
11. Cranberry Growers/Ocean Spray Scientific Advisory Board_2 days/year (honorarium)

Development of the Title, Objective, and Question

The overall objective of the research project is to systematically map when and how acceptable daily intake (ADI) values are used as health-based benchmarks in nutrition research studies that consider the safety of low-calorie sweeteners (LCS) as it relates to measured or estimated consumption. Results will be synthesized and integrated relative to the definition, development, and use of ADI values for LCS by authoritative bodies, such as the Joint FAO/WHO Expert Committee on Food Additives (JECFA), the U.S. Food and Drug Administration (FDA) or the European Food Safety Authority (EFSA).

Regarding the terminology of the type of review, it is recognized that scoping reviews (the term used in the JBI manual) are often also referred to as mapping reviews or systematic maps[3,4]. Herein, we have elected the term "systematic map" to describe the type of review as it best aligns with the overall objective.

The research question (boxed) was developed to reflect the population, concept, and context – or PCC, as described by JBI.

- Population: normal and sensitive human populations (including unhealthy populations; populations with chronic disease, etc.); any age, any sex
- Concept: comparison of ADI values established by authoritative bodies to the measured or estimated daily intake of LCS

---

[3] James et al.,2016. A methodology for systematic mapping in environmental sciences. Environmental Evidence 5:7

[4] Anderson S, Allen P, Peckham S, Goodwin N. Asking the right questions: scoping studies in the commissioning of research on the organisation and delivery of health services. Health Res Policy Syst. 2008;6(7):12

- Context: nutrition research studies; original research articles (any method) and review articles

Review Question: In nutrition research studies, how are daily intake levels of low-calorie sweeteners in human populations assessed in the context of ADIs derived by authoritative bodies?

In this mapping exercise, there are no explicit outcomes or interventions. Rather, the concept under evaluation - safe consumption levels of LCS as defined by ADI values established by authoritative bodies – implicitly contains outcomes related to safety. ADI values are unique to individual LCS, each of which is established by comprehensively reviewing toxicological data and following specific processes to conservatively establish ADI values.

Background

It is well-recognized that consumers face many challenges in understanding and applying nutritional guidance. This is especially true as consumers and health professionals alike seek to understand how to safely consume LCS due to conflicting information about safe use levels. This scenario is highlighted by a Science Advisory issued by the American Heart Association (AHA) in 2018, which reviews evidence on cardiometabolic outcomes of LCS beverages and provides recommendations related to consumption for selected populations. This includes advising against prolonged consumption of LCS by children due to a dearth of evidence on the potential adverse effects of LCS beverages relative to potential benefits (AHA, 2018)[5]. Another example is that from the American Acacemy of Pediatrics Policy Statement which also discusses the scarcity of data on long-term benefits for weight management in children and adolescents or the concsequences of long-term consumption (AAP, 2015)[6]. Such advisories may be viewed as confusing in context of the positions of authoritative bodies, such as the U.S. FDA, EFSA, or the JECFA – all of whom have established positions regarding the safety of individual LCS.

Using the U.S. FDA as an example, their website[7] clearly describes which high-intensity sweeteners are permitted for use which, "like all other ingredients added to food in the United States, must be safe for consumption." By law, the FDA undergoes a rigorous process to determine the safety of food ingredients, including the determination of the daily amount that is safe (i.e. the ADI)[8]. This involves review of scientific data by chemists, toxicologists, and other scientists to determine if there is "reasonable certainty of no harm" as defined by the laws that govern the Agency and ingredient safety requirements. As part

[5] American Heart Association (AHA). 2018. Low-Calorie Sweetened Beverages and Cardiometabolic Health – A Science Advisory From the American Heart Association. Circulation 138: e126-e140.
[6] American Academy of Pediatrics (AAP), Council on School Health, Committee on Nutrition. 2015.Snacks, Sweetened Beverages, Added Sugars, and Schools. Pediatrics: 135(3).
[7] https://www.fda.gov/Food/IngredientsPackagingLabeling/FoodAdditivesIngredients/ucm397725.htm
[8] The Food Additives Amendment of 1958 to the Federal Food, Drug, and Cosmetic Act, Pub. L. 85-929, 72 Stat. 1784 (codified as amended in 21 U.S.C. 348)

of such, many factors are considered: intake of the additive relative to the petitioned use, cumulative use/intake of the additive, and toxicological data to support the safety. Inherent to this process is the recognition that, if approved, the food additives must be safe for all populations of all ages, as well as sensitive populations (e.g., elderly, pregnant)[9].

Critical to the approval process for any authoritative body is consideration of the margin of safety. This involves a comparison of the estimated daily intake (EDI) to the acceptable daily intake (ADI). The ADI values traditionally used are those established by JECFA and other authoritative bodies, such as the U.S. FDA, Health Canada, or EFSA. ADIs are also developed using rigorous processes. As described by the World Health Organization (WHO) Environmental Health Criteria 70, "Assessing human health risks of chemicals: Derivation of guidance values for health-based exposure limits"[10], such guidance values represent estimates of intake of a substance daily over a lifetime that is considered to be without appreciable health risk. The guidance also describes how such values should be used e.g., not intended as a threshold for safety at one moment in time.

Thus, science underlying the derivation of the ADI, as well as the definition and appropriate interpretation of the ADI, are critical to enabling appropriate application when considering the safety of LCS. Recognizing the widespread consumption and global interest in LCS – particularly as they relate to public health goals of lowering added sugars as part of improving health outcomes - it is important that LCS intake be assessed appropriately relative to ADIs developed by authoritative bodies. Thus, the overarching goal of the research project is to provide a resource to assist healthcare practitioners, public health professionals, and consumers' trusted advisors in providing clarity and support for safe and appropriate use of LCS to achieve nutritional goals. By approaching the issue in a systematic manner, which involves a multidisciplinary team and reporting the resulting information as a published manuscript, the output will provide transparent, evidence-based, and peer-reviewed evidence that can be used to appropriately inform strategies for improving health outcomes.

Preliminary searches for existing scoping reviews or other evidence-based summaries did not result in any with the objectives presented herein[11]. Several recent publications that provide important context, however, were identified. For example, a review of global intakes of low/no-calorie sweeteners was published early in 2018[12]. This review examined published data on the intake of all major low-/no-calorie sweeteners and included a characterization of exposure assessment approaches. While the findings also include a comparison of findings to ADI values, this comparison was not based exclusively on that reported by the authors (which is a key differentiation with that described herein). When presenting intake estimates, Martyn et al. (2018) used "%ADI" as a metric, which could have been reported in publications or calculated by the review authors. Thus, this review provides a comprehensive summary of intake studies that will be subject to handsearching;

[9] Rulis and Levitt (2009). FDA's food ingredient approval process. Safety assurance based on scientific assessment. Regulatory Toxicology and Pharmacology 53:20-31.
[10] http://www.inchem.org/documents/ehc/ehc/ehc170.htm
[11] Searches were conducted in PROSPERO, Cochrane Database of Systematic Reviews, and PubMed
[12] Martyn et al. (2018). Low-/No-Calorie Sweeteners: A Review of Global Intakes. Nutrients, 10(3):357.

this study also serves as a publication used in validation for developing syntax for this systematic map. Additionally, a publication presenting an evidence map of LCS and health studies was identified [13]. Though this map was generated with the objective of demonstrating how an evidence-map database can be generated (using LCS and selected health outcomes as an example), it was informative to topic formulation and protocol development given the similarities approach and subject (though the concept and context were different).

Inclusion Criteria

<u>Types of Participants</u>: normal and sensitive human populations; any age, any sex

Include: studies that investigate

- Humans (any age, any sex)
- Sensitive and healthy populations
- Pregnant or nursing women
- All geographies, regions, races, and ethnicities

*Exclud*e: experimental animal studies will be excluded

<u>Concept:</u> comparison of ADI values established by authoritative bodies to measured or estimated daily intake of LCS

Include: studies that quantitatively characterize daily intake to LCS and compare such to ADI values

- LCS investigated herein include acesulfame-k, advantame, alitame, aspartame, cyclamate, d-tagatose, monk fruit, neotame, stevia, saccharin, and sucralose
    - Studies that either characterize intake to individual sweeteners, groups of sweeteners, or total/combined sweeteners will be included (though categorized as part of the map)
- Daily intake may be estimated or measured in studies via
    - Controlled exposure to LCSs
    - Cumulative or specific food intake
    - Food frequency questionnaires
    - Self-reported intake
    - Estimated dietary intake
    - Studies using blood, serum, or urinary concentrations to validate or support an estimated or controlled exposure
    - Reviews or summaries of intake as characterized by other authors
- Use or application of ADI as designated by any authoritative body (e.g., EFSA, JECFA, Scientific Committtee For Foods (SCF), FDA, Health Canada, National

[13] Wang et al., (2016). Creating a literature database of low-calorie sweeteners and health studies: evidence mapping. BMC Medical Research Methodology 16:1.

Institute of Health- National Institute of Diabetes and Digestive and Kidney Diseases (NIH-NIDDK), European Union (EU) Member states, etc.)
  - Studies comparing the level of LCS at which an effect occurs to a specified ADI

Exclude:
- Sudies estimating exposure via blood/serum or urine concentrations of metabolites of LCS
- Studies that do not provide discrete, quantitative estimates of daily consumption/intake
- Studies that do not compare intake to an ADI developed by an authoritative body or any other application of the ADI; note studies which utilize an ADI or similar type of benchmark not developed by an authoritative body will be retained for context

<u>Context:</u> nutrition research studies; original research articles (any method) and review articles

Include: Primary research, case studies/series, reviews, authoritative reports
- Studies published in any time frame

Exclude:
- Papers without title and abstract available in English
- Commentaries or editorial pieces that do not make original comparisons of intake to ADIs; such pieces may be retained for context
- Literature that is unpublished, or not peer-reviewed (i.e., grey literature with the exception of authoritative reports)

Search Strategy

The search strategy was developed by an Information Specialist (SF), informed by input from stakeholders and reviewed by the Advisory Panel. The strategy generally conforms to the three-step search strategy recommended by JBI with modifications[14] unique to the research question and underlying evidence base. The search strategy involves the following steps, both further described below:
1. Searches in two online databases (PubMed and Embase) relevant to the topic (initial and refined syntax based on a comprehensive series of validation exercises)
2. Hand-searching and reference harvesting from relevant studies and reports.
3. Targeted searching of US-FDA, EFSA, JECFA, Health Canada, Food Standards Australia New Zealand (FSANZ), and UK- Food Standards Agency (FSA)

[14] JBI recommends first, an initial search of two databases, followed by an analysis of text words from the results that are used to conduct a second search. These two steps are addressed herein via a single concatenated search based on syntax that was developed using a validation approach similar to the two-step process described by JBI.

websites and the ToxPlanet database to obtain authoritative documents related to the derivation of individual LCS ADIs.

The reviewers do not intend to contact authors of primary studies or reviews for further information.

The online database search will be facilitated by DistillerSR. Following removal of duplicates from PubMed and Embase, studies will be screened by title and abstract (TiAb) using DistillerSR. Subsequently, all studies included following the TiAb will be screened using full text to confirm inclusion.

Search syntax development and validation

Using LCS identifiers provided to the team, search syntax was developed for queries in two citation databases, PubMed and Embase. The fully developed syntax is provided below. Syntax specific to the individual sweeteners included terms to target publications using other chemical identifiers (e.g., CASRN, synonyms), trade names, and spelling variations. The second portion of the syntax consists of key words linked to food frequency intake and exposure assessments. Indexing vocabulary were included for appropriate terms in both PubMed and Embase (i.e., MeSH and Emtree, respectively).

Following initial development of syntax, search validation was performed to ensure that the syntax selection would be broad enough to capture all relevant studies (less restrictive) but narrow enough to reduce unnecessary screening (more restrictive). Based on results of the validation using previously identified publications (n=19[15]) as a proxy for efficacy, revisions to syntax were made based on terms and key words identified in studies that were not initially retrieved by the queries. Revisions to syntax increased retrieval from 58% (11/19) to 89% (17/19) and it was determined that a more restrictive search (i.e., filtered for human studies), with the addition of a very specific supplementary search captured is most appropriate for this exercise. The two remaining records (2/19) that were not retrieved evaluated plasma concentrations rather than intake, or general risk assessment methods for food additives, and thus would not be an expected result of the query.

Hand-searching, etc.

In addition to the traditional citation database search efforts, hand-searching and reference harvesting techniques will be applied. For hand-searching, high-impact journals in the field of nutrition will be identified with guidance of the Advisory Panel. The most recent edition(s) will be reviewed to identify any relevant articles which may not yet be indexed in citation databases. In regard to reference harvesting, titles in the citation list of the most

[15] Studies used for validation were identified via a series of discussions with stakeholders and experts (CD, JV) in LCS consumption and safety, as well as via secondary sources consulted as part of the problem formulation exercises.

relevant publications will be reviewed and any relevant titles will be added to the screening process.

Due to the complexity of the search, a key word feedback loop will be utilized following title and abstract screening. Studies determined to be highly relevant will be analyzed for key words and index terms, which will be compared to the original search string. If necessary, syntax will be revised, and an expanded search will be performed.

PubMed syntax:
Primary:
("Acesulfame Potassium" OR acetosulfame[Supplementary Concept] OR "Acesulfame K" OR "Ace K" OR "55589 62 3"[EC/RN number] OR Advantame OR "N-(N-(3-(3-hydroxy-4-methoxyphenyl) propyl)-alpha-aspartyl)-L-phenylalanine 1-methyl ester"[Supplementary Concept] OR "714229 20 6"[EC/RN number] OR Alitame OR "alitame" [Supplementary Concept] OR Aclame OR "80863 62 3"[EC/RN number] OR Aspartame OR Aspartame[Mesh] OR NutraSweet OR AminoSweet OR "22839 47 0"[EC/RN number] OR Canderel OR (Equal AND sweet*) OR Cyclamate OR Cyclamates[Mesh] OR "sodium n-cyclohexylsulfamate" OR "139 05 9"[EC/RN number] OR d-tagatose OR tagatose[Supplementary Concept] OR "17598 81 1"[EC/RN number] OR "Monk fruit" OR "luo han guo" OR Mogroside OR Neotame OR neotame[Supplementary Concept] OR "165450 17 9"[EC/RN number] OR "Steviol glycoside" OR steviol[Supplementary Concept] OR rebaudioside A[Supplementary Concept] OR rebaudioside C[Supplementary Concept] OR rebaudioside D[Supplementary Concept] OR rebaudioside E[Supplementary Concept] OR Rebaudioside OR "Dulcoside A" OR dulcoside A[Supplementary Concept] OR Stevioside OR stevioside[Supplementary Concept] OR Stevia OR Stevia[Mesh] OR Saccharin OR Saccharin[Mesh] OR "Benzoic sulfimide" OR "81 07 2"[EC/RN number] OR trichlorosucrose[Supplementary Concept] OR Sucralose OR "56038 13 2"[EC/RN number] OR Splenda OR "56038 13 2"[EC/RN number] OR "Sweet'n Low" OR Truvia OR ((sweetener OR sweeteners) AND ((high AND intensity) OR high-intensity OR "sugar free" OR "sugar-free" OR "table-top" OR "table top" OR "intense" OR "artificial" OR "low calorie" OR low-calorie OR "no calorie" OR no-calorie OR nonnutritive OR non-nutritive OR noncaloric OR non-caloric) OR LNCS[tiab] OR "low and no calorie sweeteners" OR "Non-Nutritive Sweeteners"[Mesh]) AND ("post marketing surveillance" OR "postmarketing surveillance" OR "post-marketing surveillance" OR ((estimated OR anticipated) AND (exposure OR intake)) OR "Dietary Exposure"[Mesh] OR "dietary exposure" OR "dietary intake" OR "Eating"[Mesh] OR "acceptable daily intake" OR "No-Observed-Adverse-Effect Level"[Mesh] OR "Recommended Dietary Allowances"[Mesh] OR "food consumption" OR "exposure assessment" OR ((consumption OR dietary OR food) AND (frequency OR data OR questionnaire OR survey))) AND "humans"[MeSH]

Supplementary:
("Acesulfame Potassium" OR acetosulfame[Supplementary Concept] OR "Acesulfame K" OR "Ace K" OR "55589 62 3"[EC/RN number] OR Advantame OR "N-(N-(3-(3-hydroxy-4-methoxyphenyl) propyl)-alpha-aspartyl)-L-phenylalanine 1-methyl ester"[Supplementary Concept] OR "714229 20 6"[EC/RN number] OR Alitame OR

"alitame" [Supplementary Concept] OR Aclame OR "80863 62 3"[EC/RN number] OR Aspartame OR Aspartame[Mesh] OR NutraSweet OR AminoSweet OR "22839 47 0"[EC/RN number] OR Canderel OR (Equal AND sweet*) OR Cyclamate OR Cyclamates[Mesh] OR "sodium n-cyclohexylsulfamate" OR "139 05 9"[EC/RN number] OR d-tagatose OR tagatose[Supplementary Concept] OR "17598 81 1"[EC/RN number] OR "Monk fruit" OR "luo han guo" OR Mogroside OR Neotame OR neotame[Supplementary Concept] OR "165450 17 9"[EC/RN number] OR "Steviol glycoside" OR steviol[Supplementary Concept] OR rebaudioside A[Supplementary Concept] OR rebaudioside C[Supplementary Concept] OR rebaudioside D[Supplementary Concept] OR rebaudioside E[Supplementary Concept] OR Rebaudioside OR "Dulcoside A" OR dulcoside A[Supplementary Concept] OR Stevioside OR stevioside[Supplementary Concept] OR Stevia OR Stevia[Mesh] OR Saccharin OR Saccharin[Mesh] OR "Benzoic sulfimide" OR "81 07 2"[EC/RN number] OR trichlorosucrose[Supplementary Concept] OR Sucralose OR "56038 13 2"[EC/RN number] OR Splenda OR "56038 13 2"[EC/RN number] OR "Sweet'n Low" OR Truvia OR ((sweetener OR sweeteners) AND ((high AND intensity) OR high-intensity OR "low calorie" OR low-calorie OR "no calorie" OR no-calorie OR nonnutritive OR non-nutritive OR noncaloric OR non-caloric) OR LNCS[tiab] OR "low and no calorie sweeteners" OR "Non-Nutritive Sweeteners"[Mesh]) AND ("acceptable daily intake" OR "ADI")

Embase Syntax
Primary:
'acesulfame potassium' OR 'acesulfame'/exp OR 'acesulfame k' OR 'ace k' OR '55589 62 3':rn OR advantame OR 'advantame'/exp OR '714229 20 6':rn OR alitame OR 'alitame'/exp OR aclame OR '80863 62 3':rn OR aspartame OR 'aspartame'/exp OR nutrasweet OR aminosweet OR '22839 47 0':rn OR canderel OR (equal AND sweet*) OR cyclamate OR 'cyclamate sodium'/exp OR 'sodium n-cyclohexylsulfamate' OR '139 05 9':rn OR 'd tagatose' OR 'tagatose'/exp OR '17598 81 1':rn OR 'monk fruit' OR 'luo han guo' OR mogroside OR 'mogroside'/exp OR neotame OR 'neotame'/exp OR '165450 17 9':rn OR 'steviol glycoside' OR 'steviol glycoside'/exp OR 'rebaudioside a'/exp OR 'rebaudioside b'/exp OR 'rebaudioside c'/exp OR 'rebaudioside d'/exp OR 'rebaudioside e'/exp OR 'rebaudioside'/exp OR rebaudioside OR 'dulcoside a' OR 'dulcoside a'/exp OR stevioside OR 'stevioside'/exp OR stevia OR 'stevia'/exp OR saccharin OR 'saccharin'/exp OR 'benzoic sulfimide' OR '81 07 2':rn OR 'sucralose'/exp OR sucralose OR splenda OR '56038 13 2':rn OR 'sweet n low' OR truvia OR lncs:ti,ab OR 'low and no calorie sweeteners' OR 'nonnutritive sweetener'/exp OR ((sweetener OR sweeteners) AND ('high intensity' OR 'low calorie' OR 'no calorie' OR nonnutritive OR 'non nutritive' OR noncaloric OR 'non caloric')) AND ('post marketing surveillance' OR 'postmarketing surveillance' OR 'post-marketing surveillance' OR 'postmarketing surveillance'/exp OR ((estimated OR anticipated) AND (exposure OR intake)) OR 'dietary exposure'/exp OR 'dietary exposure' OR 'dietary intake' OR 'food intake'/exp OR 'dietary reference intake'/exp OR 'acceptable daily intake'/exp OR 'acceptable daily intake' OR 'food consumption' OR 'exposure assessment' OR 'exposure assessment'/exp OR 'food frequency questionnaire'/exp OR ((consumption OR dietary OR food) AND (frequency OR data OR questionnaire OR survey)))

Supplementary:
('acesulfame potassium' OR 'acesulfame'/exp OR 'acesulfame k' OR 'ace k' OR '55589 62 3':rn OR advantame OR 'advantame'/exp OR '714229 20 6':rn OR alitame OR 'alitame'/exp OR aclame OR '80863 62 3':rn OR aspartame OR 'aspartame'/exp OR nutrasweet OR aminosweet OR '22839 47 0':rn OR canderel OR (equal AND sweet*) OR cyclamate OR 'cyclamate sodium'/exp OR 'sodium n-cyclohexylsulfamate' OR '139 05 9':rn OR 'd tagatose' OR 'tagatose'/exp OR '17598 81 1':rn OR 'monk fruit' OR 'luo han guo' OR mogroside OR 'mogroside'/exp OR neotame OR 'neotame'/exp OR '165450 17 9':rn OR 'steviol glycoside' OR 'steviol glycoside'/exp OR 'rebaudioside a'/exp OR 'rebaudioside b'/exp OR 'rebaudioside c'/exp OR 'rebaudioside d'/exp OR 'rebaudioside e'/exp OR 'rebaudioside'/exp OR rebaudioside OR 'dulcoside a' OR 'dulcoside a'/exp OR stevioside OR 'stevioside'/exp OR stevia OR 'stevia'/exp OR saccharin OR 'saccharin'/exp OR 'benzoic sulfimide' OR '81 07 2':rn OR 'sucralose'/exp OR sucralose OR splenda OR '56038 13 2':rn OR 'sweet n low' OR truvia OR lncs:ti,ab) AND ('acceptable daily intake' OR 'ADI')

Extraction of the Results

The review team will chart results via a project-specific form developed in DistillerSR, following the framework for scoping reviews as described by JBI. Briefly, key information such as citation details (e.g., author, year), study details (e.g., objective, population, methods), quantitative or qualitative results as related to the application of an ADI to LCS exposures, and strengths and limitations of the studies will be documented. If possible, details regarding geographical region and ADI authority (e.g., EFSA, JECFA, US-FDA) will also be collected. Following the development of a draft form, the review team will pilot extraction to ensure reviewer consistency and to identify any additional data determined to be useful to informing the research question. The pilot exercises may result in iterative refinement of the DistillerSR form. All information from DistillerSR will be exported into Microsoft Excel to develop tabular summaries for distribution, synthesis, and reporting.

Presentation of Results

Tabular summaries and data visualizations of the extracted information will be constructed as warranted to characterize the landscape map of how ADI information is used in context of studies which consider the safety of LCS. It is anticipated that maps will be developed for individual sweeteners; as part of such the ADI value utilized will be mapped as well as to how it was used relative to the populations of interest. Maps will help facilitate synthesis and determination of how ADI values are being applied, and if they are being utilized in a manner consistent with the prescribed definition, development, and use of ADI values for LCS by authoritative bodies, such as JECFA, the US-FDA or EFSA. It is anticipated that findings will also be discussed in context of selected public-facing sources, such as websites from WebMD, the Mayo Clinic, AHA, American Diabetes Association (ADA), etc. Key findings, data gaps, and future research recommendations will be developed narratively and supported by the tabular summaries and data visualizations.

The systematic map will be reported in a manuscript that will be submitted to a peer-review journal. The goal of the manuscript will be to both report the systematic map with discussion aimed at increasing clarity surrounding the derivation and intended use of ADIs. It is anticipated that the manuscript will contain background on the history of deriving an ADI (including lifetime daily average intakes, life stages, risk assessment context of intakes) in addition to the systematic map findings. The target audience includes researchers, clinicians and dietitians, and other practitioners who may consult ADIs when providing nutritional advice to individuals.
